# Human IL-6 fosters long-term engraftment of patient-derived disease-driving myeloma cells in immunodeficient mice

**DOI:** 10.1172/jci.insight.177300

**Published:** 2024-05-07

**Authors:** Zainul S. Hasanali, Alfred L. Garfall, Lisa Burzenski, Leonard D. Shultz, Yan Tang, Siddhant Kadu, Neil C. Sheppard, Wei Liu, Derek Dopkin, Dan T. Vogl, Adam D. Cohen, Adam J. Waxman, Sandra P. Susanibar-Adaniya, Martin Carroll, Edward A. Stadtmauer, David Allman

**Affiliations:** 1 Division of Hematology Oncology, University of Pennsylvania, Philadelphia, Pennsylvania, USA.; 2 Jackson Laboratories, Bar Harbor, Maine, USA.; 3 Center for Cellular Immunotherapies,; 4 Department of Pathology and Laboratory Medicine, and; 5 Stem Cell and Xenograft Core Facility, Perelman School of Medicine, University of Pennsylvania, Philadelphia, Pennsylvania, USA.

**Keywords:** Hematology, Bone marrow, Cancer, Mouse models

## Abstract

Multiple myeloma is a largely incurable and life-threatening malignancy of antibody-secreting plasma cells. An effective and widely available animal model that recapitulates human myeloma and related plasma cell disorders is lacking. We show that busulfan-conditioned human IL-6–transgenic (hIL-6–transgenic) NSG (NSG+hIL6) mice reliably support the engraftment of malignant and premalignant human plasma cells, including from patients diagnosed with monoclonal gammopathy of undetermined significance, pre- and postrelapse myeloma, plasma cell leukemia, and amyloid light chain amyloidosis. Consistent with human disease, NSG+hIL6 mice engrafted with patient-derived myeloma cells developed serum M spikes, and a majority developed anemia, hypercalcemia, and/or bone lesions. Single-cell RNA sequencing showed nonmalignant and malignant cell engraftment, the latter expressing a wide array of mRNAs associated with myeloma cell survival and proliferation. Myeloma-engrafted mice given CAR T cells targeting plasma cells or bortezomib experienced reduced tumor burden. Our results establish NSG+hIL6 mice as an effective patient-derived xenograft model for study and preclinical drug development of multiple myeloma and related plasma cell disorders.

## Abstract 2

Busulfan-conditioned human IL-6-transgenic NSG mice support the engraftment of malignant and premalignant human plasma cells, providing an effective patient-derived xenograft multiple myeloma model.

## Introduction

Multiple myeloma (MM) and related clonal bone marrow (BM) plasma cell dyscrasias (PCDs) cause approximately 100,000 deaths per year worldwide (1). In addition to MM, these disorders include a premalignant state called monoclonal gammopathy of undetermined significance (MGUS) (2), a highly aggressive and therapy-resistant leukemia termed plasma cell leukemia (PCL) (3), and amyloid light chain (AL) amyloidosis, which is characterized by the formation of monoclonal antibody–driven amyloid fibrils (4). Despite substantial recent advances in therapeutic options for patients with MM, PCL, and AL amyloidosis that build on the previous success of proteasome inhibitors and thalidomide analogs (5, 6), the majority of patients experience relapse and eventually succumb to complications of treatment-refractory disease (7).

A major roadblock to curative drug development for MM and other PCDs has been the lack of a flexible and readily accessible animal model that recapitulates human disease. In principle, any such model would support the long-term persistence and growth of primary patient-derived PCDs in a manner that mirrors both the growth properties of PCDs and key clinical signs such as anemia, hypercalcemia, renal damage, and bone destruction. Currently, a common approach to study novel therapeutics in vivo is in immunodeficient mice engrafted with MM cell lines (8–10). However, cell line xenograft models fail to reliably recapitulate many aspects of clinical disease, do not faithfully model drug resistance mechanisms, and the cell lines used often grow aggressively, in contrast with most slower growing PCDs (11, 12). An alternative approach involves engraftment of human fetal or rabbit bone chips implanted into immunodeficient mice; however, these systems fail to drive clinical signs of disease, and the bone-resident MM cells do not disseminate throughout the skeleton, as the disease does in humans (13).

Two patient-derived xenograft models have been reported for primary myeloma. The first uses NSG mice in a similar approach to that presented here (14). However, prolonged engraftment, characterization of engrafted cells, characterization of clinical phenotypes, and evaluation of cellular immunotherapies have not been performed. The second, from Das et al., showed that immunodeficient mice (RAG2^–/–^ γc^–/–^) harboring humanized versions of several cytokines, including G-CSF, GM-CSF, IL-3, and IL-6 (MISTRG6 mice) afford robust engraftment of patient PCDs (15). Although Das et al. determined that IL-6 is essential for PCD engraftment, the necessity of the other humanized genes was not firmly established. This more complex model is also difficult to obtain and requires continuous antibiotic administration, resulting in limited use within the myeloma research community.

We studied the engraftment and long-term persistence of all major PCDs after transfer into transgenic NSG mice harboring a bacterial artificial chromosome (BAC) containing the human IL-6 gene (NSG+hIL6). We reasoned that increasing systemic IL-6 levels with a humanized BAC might be advantageous, because the BAC is likely to contain *cis* regulatory elements needed for proper cell-type-restricted IL-6 expression and because mouse IL-6 does not stimulate the human IL-6 receptor (16). Our results establish NSG+hIL6 mice as a straightforward and readily accessible system for the study of a wide range of PCD disease manifestations and therapies, including newly diagnosed and relapsed myeloma.

## Results

**Figure 1:**
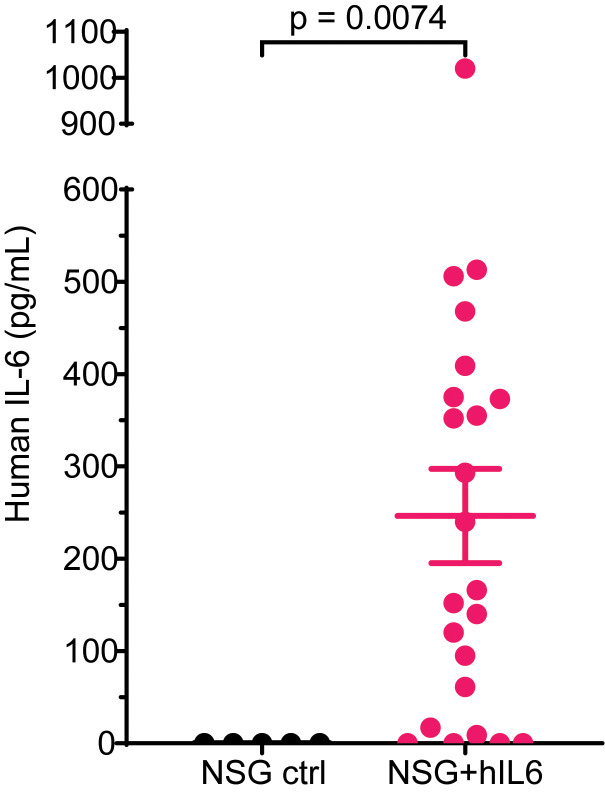
Human IL-6 in NSG+hIL6 sera. Sera from 12- to 20-week-old NSG (*n* = 5) and NSG+hIL6 (*n* = 23) mice were evaluated for human IL-6 levels by quantitative ELISA. Horizontal lines and error bars indicate the mean and the standard deviation of the mean, respectively. Statistics were calculated with the Kolmogorov-Smirnov comparison.

NSG+hIL6 transgenic mice were generated by microinjecting a BAC containing the promoter and gene elements of the human IL-6 gene on chromosome 7 into fertilized embryos of NOD.CB17-*Prkdc^scid^*/J mice, and the resulting mice were crossed to eventually generate NSG+hIL6 mice (see Methods) (17). Because heterozygous females had low fertility, we bred males with normal NSG females; approximately 50% of the resulting pups carried the BAC. ELISA analyses showed that the majority of NSG+hIL6 mice possessed hIL-6 (mean 246.3 pg/mL, range 0–1020 pg/mL) in sera. hIL-6 levels distributed into 2 groups: 9–300 pg/mL and 300–600 pg/mL (Figure 1). There were no associations or trends observed in downstream experiments between the 2 groups. These IL-6 levels were higher than those observed in normal human sera (<5 pg/mL) (18), yet they were in the range of IL-6 expression in PCDs (0.01–4 ng/mL) (19).

### Patient MM cell engraftment in NSG+hIL6 mice.

**Figure 2:**
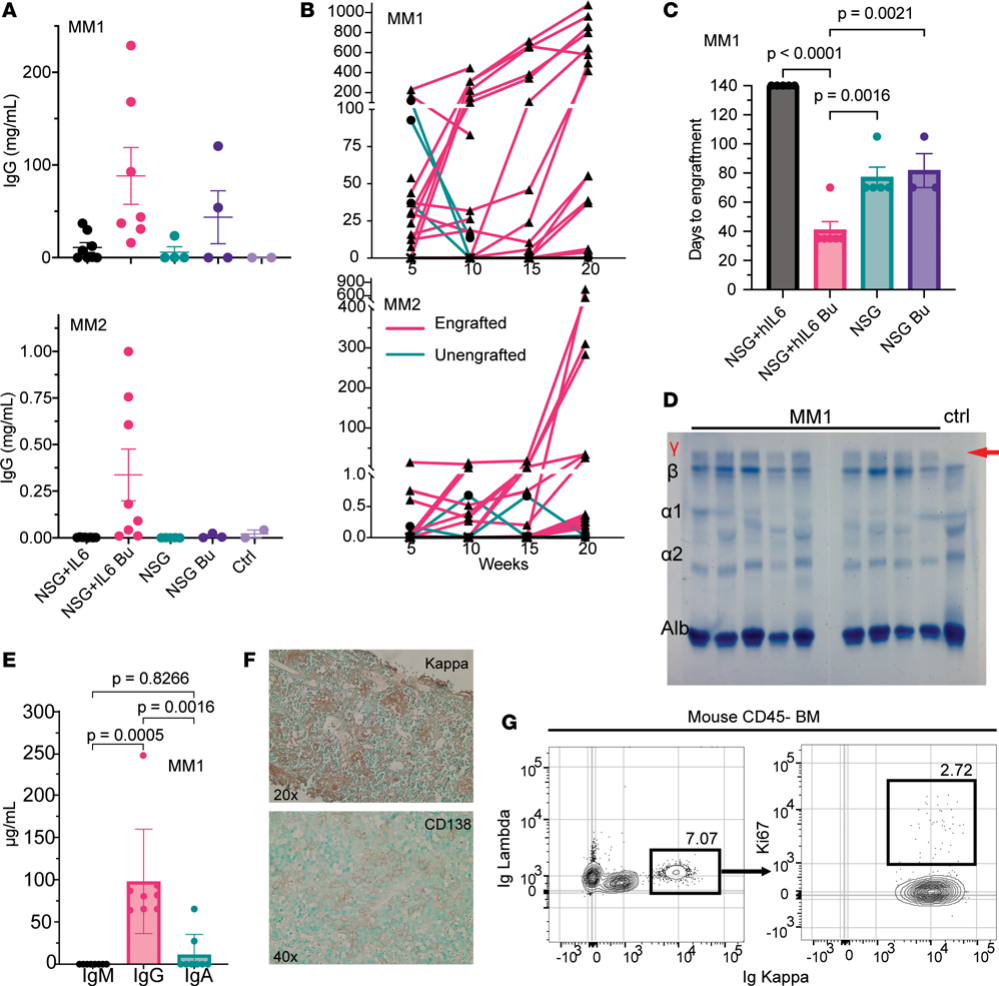
NSG+hIL6 mice support primary patient MM. BM cells from 1 of 2 newly diagnosed MM patients (MM1 and MM2) were transferred via intraosseous injection into NSG or NSG+hIL6 adults with and without busulfan pretreatment. MM1: NSG+hIL6 (*n* = 8), NSG+hIL6 busulfan (*n* = 7), NSG (*n* = 4), NSG busulfan (*n* = 4); MM2: NSG+hIL6 (*n* = 7), NSG+hIL6 busulfan (*n* = 8), NSG (*n* = 5), NSG busulfan (*n* = 3); No myeloma control (*n* = 2). (**A**) Sera from the indicated cohorts were evaluated for human IgG levels by ELISA 5 weeks after injection. “Ctrl” indicates saline-injected NSG+hIL6 mice. Horizontal lines and error bars indicate the mean and standard deviation of the mean, respectively. (**B**) Serum IgG levels in MM1- and MM2-engrafted mice over 20 weeks grouped by engraftment status (unengrafted: green line and circle; engrafted: pink line and triangle). (**C**) Time to detection of serum human Ig (functional engraftment) for NSG versus NSG+hIL6 hosts with or without preconditioning with MM1 (NSG+hIL6 busulfan vs. NSG [*P* = 0.0016], NSG+hIL6 [*P* < 0.0001], or NSG busulfan [*P* = 0.0021]). Each data point represents a single mouse. (**D**) SPEP analysis (*n* = 9) of sera samples from mice engrafted with MM1 versus an unengrafted control (*n* = 1). Gamma region denoted with the red γ. Red arrow denotes M spike representative of myeloma engraftment. (**E**) Total IgM, IgG, and IgA serum levels (*n* = 7) from mice engrafted with MM1 were determined by ELISA. (**F**) Histologic sections prepared from the BM of an MM1-engrafted NSG+hIL6 host were stained with antibodies specific for human Igκ or CD138. (**G**) BM cells from an MM1 engrafted NSG+hIL6 host were pregated on viable mouse CD45^–^ and human CD3^–^CD20^–^ cells evaluated for intracellular Igκ and Igλ and Ki67 expression. Statistics for **C** and **E** were calculated using Dunnett’s multiple-comparison test and Tukey’s multiple-comparison test, respectively. Results in **F** are representative of similarly observed findings from 12 mice.

Using established xenotransplantation protocols (20), we examined the impact of host preconditioning with busulfan with and without the presence of the human *IL6* locus on engraftment of primary MM cells following intraosseous injection of T cell–depleted patient BM mononuclear cells. Initially, we tested for engraftment of malignant plasma cells from 2 newly diagnosed MM patients (MM1 and MM2) following transfer of 1 × 10^6^ mononuclear BM cells per mouse. We evaluated human antibodies in sera every 5 weeks out to 20 weeks after transfer and then at 52 weeks after transfer. Within 5 weeks we readily detected human Ig in sera in busulfan-treated NSG+hIL6 mice for both MM1 and MM2. By contrast, at this time, engraftment was far less routine for busulfan-treated NSG mice and NSG+hIL6 mice without busulfan (Figure 2A). Furthermore, for most preconditioned NSG+hIL6 mice, serum titers for human Ig increased progressively over 20 weeks (Figure 2B). Time to engraftment was defined by the initial detection of human Ig in mouse sera. Whereas the majority of NSG+hIL6 mice exhibited clear signs of engraftment within 5–10 weeks, by 20 weeks after injection a much smaller fraction of NSG mice scored positive for human Ig serum antibodies, and surprisingly time to engraftment was especially prolonged for nonconditioned NSG+hIL6 hosts (Figure 2C). Although small numbers of human T cells were detected by single-cell RNA-seq (scRNA-seq) (see below), we did not observe clinical signs of graft-versus-host disease (GvHD) in any mice. Serum protein electrophoresis (SPEP) gels revealed a gamma region M spike for 9 tested xenografted mice at 15 weeks after injection that was absent in a non-xenografted control (Figure 2D). Also consistent with engraftment of monoclonal plasma cells, ELISA for human heavy chain IgG, IgM, or IgA showed the presence of only IgG (Figure 2E). Staining of BM tissue sections with anti–human CD138 and kappa light chain antibodies revealed clusters of light chain–restricted human plasma cells (Figure 2F). Flow cytometric analyses of BM cells from serum IgG^+^ NSG+hIL6 mice implanted from MM1 showed Igκ-restricted light chain expression (Figure 2G), in line with the engrafted myeloma clone. The fraction of all BM cells that were human myeloma cells ranged from less than 1% to 12% (±4%). Consistent with the slow growth rate of malignant plasma cells, less than 3% of myeloma cells derived from MM2-engrafted mice were Ki67^+^ (21) (Figure 2G). We concluded that NSG+hIL6 mice with busulfan conditioning were superior in providing a supportive environment for the efficient engraftment and long-term persistence of primary MM cells. Therefore, we used busulfan-preconditioned NSG+hIL6 mice for all subsequent experiments.

### Engraftment of a spectrum of PCDs.

**Figure 3:**
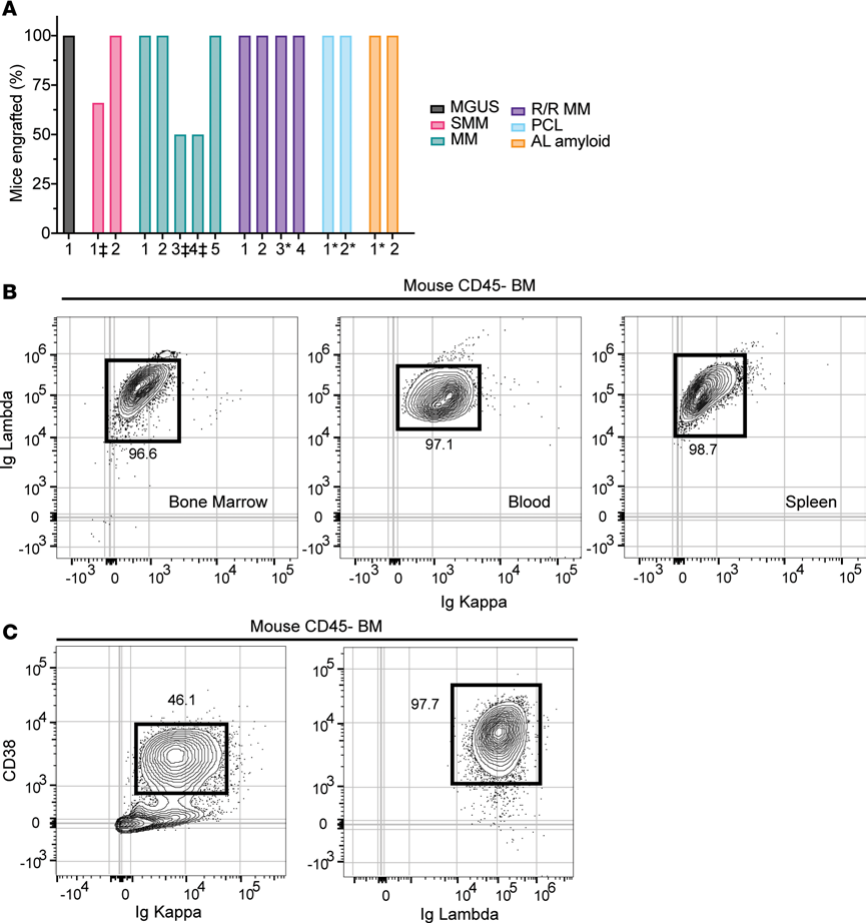
NSG+hIL6 mice support major plasma cell dyscrasias. NSG+hIL6 mice served as hosts for BM cells derived from patients with MGUS (*n* = 1), smoldering multiple myeloma (SMM) (*n* = 2), newly diagnosed multiple myeloma (MM) (*n* = 5), relapsed/refractory myeloma (R/R MM) (*n* = 4), plasma cell leukemia (PCL) (*n* = 2), or AL amyloidosis (AL amyloid) (*n* = 2). (**A**) Shown is the fraction of mice in each cohort with sera scoring positive for human IgG patients at 10 weeks after transfer (*n* = 5 hosts/group). *Recipients of previously frozen human BM cells. ‡Samples not reaching 100% engraftment were prematurely terminated after 3 weeks due to mycoplasma contamination. (**B**) Flow cytometric analysis for Ig lambda and Ig kappa expression in permeabilized mouse BM (left), blood (middle), and spleen (right) cells harvested from an NSG+hIL6 mouse engrafted with BM from a PCL patient. (**C**) Analysis of CD38 and Ig kappa or Ig lambda expression for mouse BM cells from separate NSG+hIL6 hosts engrafted previously with BM cells from the MM2 donor (left) or the PCL patient presented in **B**. For **B** and **C**, plots were gated on viable mouse CD45^–^ singlets.

Next, we asked whether busulfan-preconditioned NSG+hIL6 mice support engraftment of other PCDs. With the exception of 3 samples that were excluded early due to sample mycoplasma contamination, we were able to engraft 100% of NSG+hIL6 mice with 100% of samples from donors experiencing MGUS, smoldering MM, de novo MM, relapsed/refractory (R/R) MM, PCL, and AL amyloidosis (Figure 3A). This included 100% engraftment of all NSG+hIL6 mice from 3 cryopreserved relapsed MM or PCL patients from 5 years earlier (Figure 3A, asterisks). The ability to use cryopreserved specimens increases the potential use of this model outside of primary myeloma referral centers.

Flow cytometric analysis of BM from Igλ^+^ PCL–engrafted mice showed Igλ-restricted light chain expression by the BM engrafted clone (Figure 3B). Additionally, Igλ^+^-restricted cells dominated the blood (Figure 3B, middle panel) and were noted in spleen (Figure 3B). Circulating disease was only detectable in mice engrafted with BM cells from a PCL patient, not other PCDs, in line with observed PCL human phenotypes. Also of note, whereas we often detected surface expression of the ectoenzyme and drug target CD38, its levels varied on the plasma cells derived from different donors (Figure 3C). We conclude that the BM microenvironment of NSG+hIL6 mice supports the engraftment of a wide variety of PCDs with similar disease-affiliated characteristics as their human donors.

### scRNA-seq analyses.

**Figure 4:**
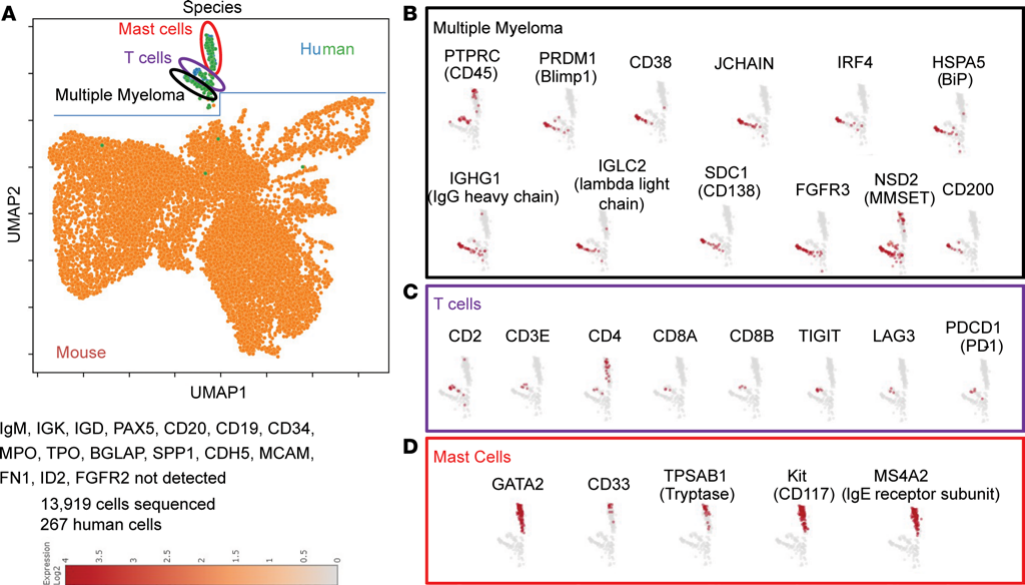
Characterization of NSG+hIL6 myeloma-engrafted mice. BM from an NSG+hIL6 mouse engrafted with mononuclear human BM cells from patient sample R/R MM3 was isolated 52 weeks after intraosseous injection and subjected to scRNA-seq using the Parse Biosciences processing and analysis pipeline. (**A**) Uniform manifold approximation and projection (UMAP) denotes the presence of mouse cells (orange) and human cells (green and blue). Human cells form 3 clusters. Gene expression profiles define these as (**B**) myeloma cells (black), (**C**) T cells (purple), and (**D**) mast cells (red). The data herein represent scRNA-seq from 1 mouse engrafted with 1 human MM sample.

Because we engrafted unsorted BM mononuclear cells from patients with PCDs, we sought to further characterize human cells engrafted into NSG+hIL6 hosts. We performed scRNA-seq on total BM cells from an NSG+hIL6 mouse 52 weeks after implantation with mononuclear BM cells from a patient with IgG lambda R/R MM with t(4;14), sample MM3. We utilized the Parse Biosciences pipeline to prepare and analyze data. Human and mouse cells were distinguished by the presence of species-specific mRNA transcripts. As shown in blue and green (Figure 4), human cells comprised a small fraction of total BM cells and segregated into 3 clusters. These cells included a cluster containing clonal human plasma cells denoted by mRNAs for the IGHG1 and IGL2 genes, the myeloma and plasma cell transcription factors BLIMP1 (22) and IRF4 (23), and the myeloma-associated proteins CD38 (24), CD200 (25), fibroblast growth factor receptor 3 (FGFR3), and nuclear receptor binding SET domain protein 2 (NSD2) (26), the latter 2 resulting from the t(4;14) translocation present in this patient’s myeloma. Additionally, we detected human T cells (CD2^+^CD3ε^+^) and mast cells (c-Kit^+^GATA2^+^, IgE Fc receptor subunit β^+^). T cells were enriched for transcripts for immune quiescence (TIGIT, LAG3, PD1), and, notably, no GvHD was observed. No human CD34^+^ stem cell, B cell (IgM, IgD, PAX5, CD20, CD19), macrophage (CD16, CD14), neutrophil (MPO), megakaryocyte (TPO), stromal cell (FN1, FGFR2), osteoblast (BGLAP, SPP1), eosinophil (ID2), or endothelial cell (CDH5, MCAM) specific markers were detected, arguing against routine engraftment of hematopoietic stem cells. Based on the results in Figures 1–4, we conclude that NSG+hIL6 mice support the efficient and long-term engraftment of primary PCDs.

### Myeloma-engrafted NSG+hIL6 mice exhibit signs of disease.

**Figure 5:**
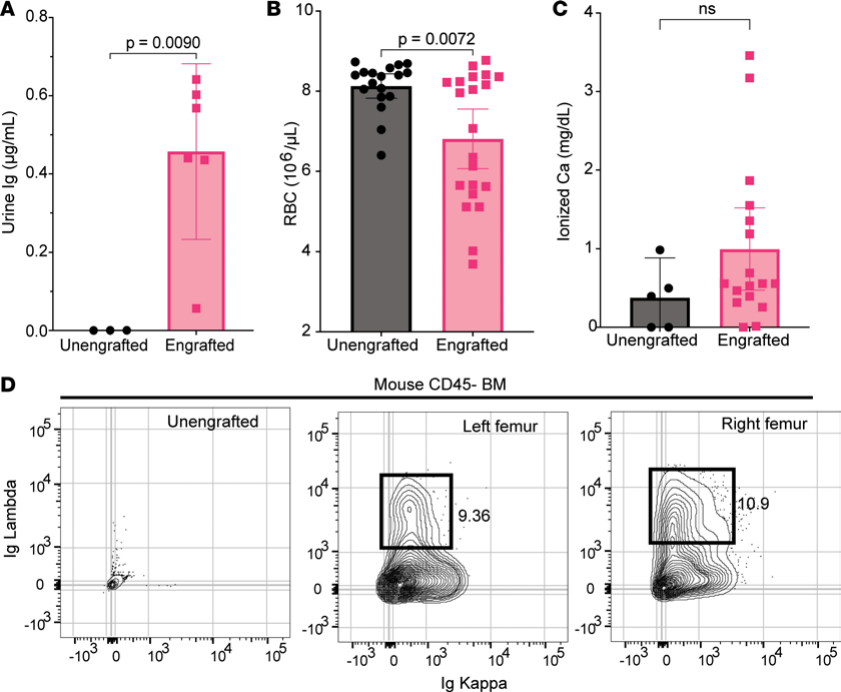
Myeloma-engrafted NSG+hIL6 mice with sequelae of human disease. (**A**) Urine from NSG+hIL6 mice (*n* = 6) engrafted 15 weeks previously with MM1 BM cells and unengrafted controls (*n* = 3) was evaluated for human Ig. (**B**) RBC counts from engrafted (*n* = 18) versus unengrafted (*n* = 21) mice at 15 weeks after injection. (**C**) Serum ionized calcium concentrations in engrafted (*n* = 15) compared with unengrafted controls (*n* = 5) at 15 weeks. (**D**) Flow cytometric analysis of Ig kappa and Ig lambda expression for permeabilized BM cells from an unengrafted NSG+hIL6 mouse (left plot), the left femur (middle plot), and right femur (right plot) of a serum human IgG^+^ NSG+hIL6 mouse given MM1 BM cells 12 weeks previously. BM cells were only injected into the left femur. Columns and error bars indicate the mean and standard deviation of the mean, respectively. Statistics were calculated with 2-tailed Mann-Whitney *t* tests. Flow cytometric images in **D** are representative of 12 mice with similar findings.

To test the utility of NSG+hIL6 mice for study of MM-associated disease states, we probed for signs of urine Ig, anemia, hypercalcemia, MM cell dissemination throughout the skeleton, and bone destruction in mice engrafted with cells from the MM1 or MM2 donor. Due to logistic reasons, not all mice were able to be tested for all clinical sequelae of disease. At 15 weeks after injection, urine from several engrafted mice possessed detectable titers of human Ig (Figure 5A), similar to many MM patients. Likewise, RBC counts were significantly lower in serum IgG^+^ mice compared with nonengrafted controls (Figure 5B). Third, although not common, mice with ionized serum calcium levels were detected in IgG^+^ mice at levels well above those of nonengrafted mice (Figure 5C). Fourth, whereas all mice were inoculated in their left femur, at 8 weeks after transfer, Igκ^+^ MM cells were readily detected in both the left (Figure 5D) and the right femur (Figure 5D), confirming spread within the skeleton, a hallmark of MM.

**Figure 6:**
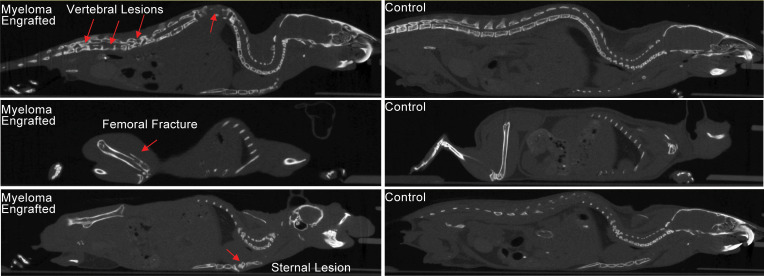
Myeloma-engrafted NSG+hIL6 mice develop skeletal lesions. MicroCT scans of surviving human IgG^+^ NSG+hIL6 mice were performed at 52 weeks after injection. Vertebral (top left), femoral (middle left), and sternal (bottom left) lytic lesions in MM1- and MM2-engrafted mice (red arrows) were noted compared with NSG+hIL6 mice not engrafted with MM. Lesions are representative of 14 imaged animals.

**Figure 7:**
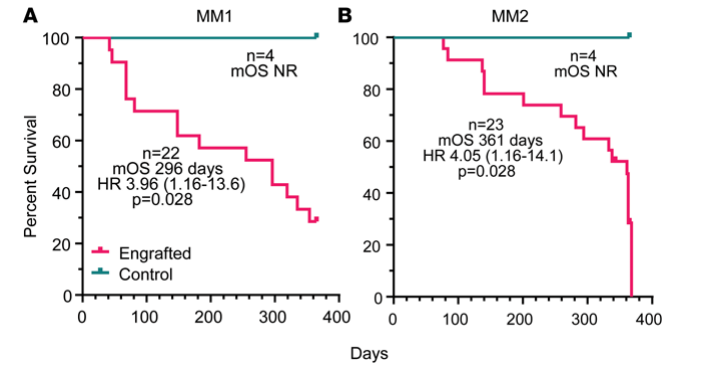
Mortality of myeloma-engrafted NSG+hIL6 mice. Kaplan-Meier curves for NSG+hIL6 mice that were engrafted at 16 weeks of age with BM cells from donor MM1 (engrafted, *n* = 22; unengrafted, *n* = 4) (**A**) or MM2 (engrafted, *n* = 23; unengrafted, *n* = 4) (**B**). All mice were monitored for humane endpoints over the indicated time frames. There was only a single mouse, within the MM1 cohort, that was injected with myeloma cells and died before IgG was detectable in the serum. All others had detectable IgG at the time of death. Statistics were calculated with log-rank (Mantel-Cox) testing. mOS, mean overall survival; HR, hazard ratio; NR, not reached.

At 52 weeks, several engrafted mice were assessed for skeletal abnormalities by microCT scan prior to euthanasia. These mice showed thinned bone with vertebral lesions, sternal lesions, and even a fractured femur (Figure 6). All of these clinical manifestations are commonly observed in advanced human myeloma (27). Together, these data indicate that the NSG+hIL6 xenograft model also recapitulates the clinical sequelae of human MM, a feature that heretofore has not been described in other models. Lastly, the majority of engrafted mice succumbed between approximately 100 and 400 days after transfer and eventually all mice died. Except for one mouse, all mice died only after detection of circulating Ig, indicating myeloma was responsible for death. The median overall survival of MM1 and MM2 was 296 and 361 days, respectively (Figure 7). When cause of death was analyzed, 11 (24%) mice had hind limb paralysis, 16 (35%) became moribund, and 14 (30%) were found dead in their cage (Table 1 and Table 2).

### Responses to antimyeloma therapies.

**Figure 8:**
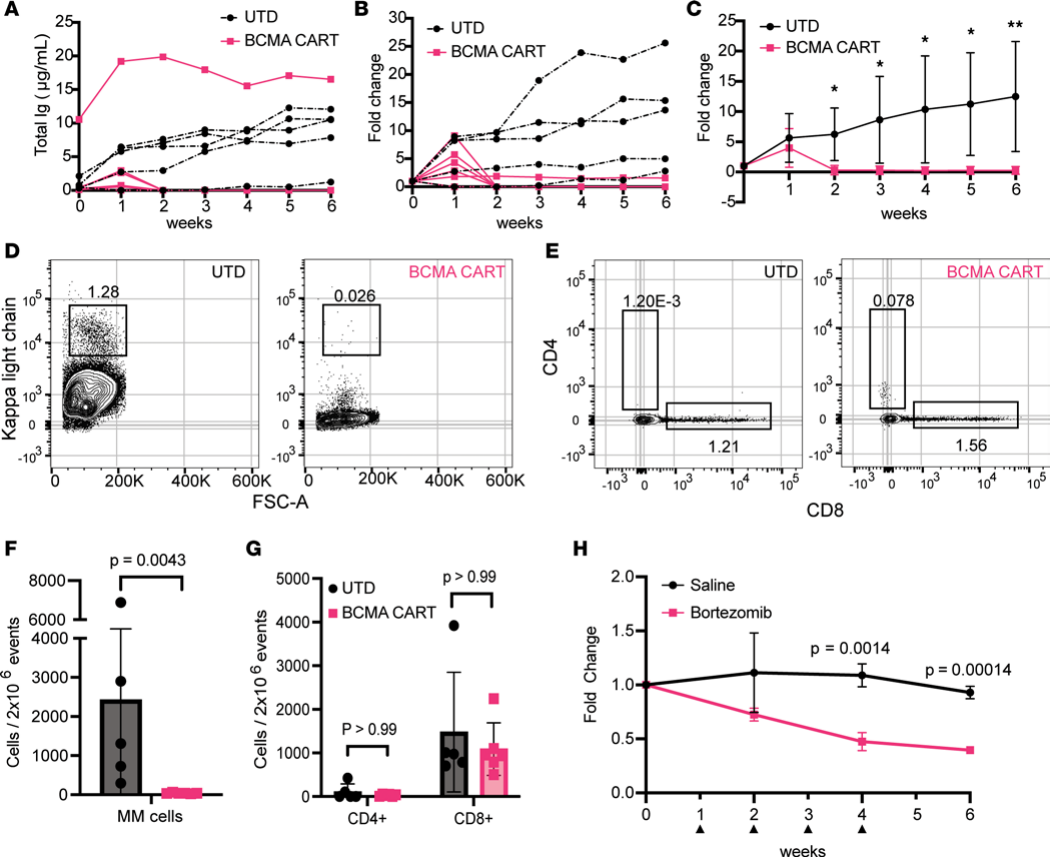
Responses to BCMA-CART or bortezomib. (**A**–**G**) NSG+hIL6 mice were implanted with BM cells from an untreated newly diagnosed Igκ^+^ MM patient. Fourteen weeks later (week zero), serum Ig^+^ mice were given a single dose of human BCMA-CART cells (pink, *n* = 6) or untransduced T cells (UTD) from the same normal donor (black, *n* = 5). Sera were analyzed by ELISA for human Ig weekly over 6 weeks (**A**–**C**). Shown are total Ig levels (**A**), fold change over time (**B**), and aggregate fold change data for each group (**C**). *P < 0.05, **P < 0.005. For **A** and **B**, each line derives from an individual host. (**D** and **E**) Representative flow cytometric plots on week 6 for Igκ^+^ (**D**) or CD4^+^ and CD8^+^ T cells (**E**) among BM cells in recipients of UTD or BCMA-CART cells as indicated. Igκ-versus-FSC plots are pregated on viable CD19^–^ and CD3^–^ events; CD4^+^ versus CD8^+^ T cell plots are pregated on viable CD3^+^ events. (**F** and **G**) Means and standard deviations for Igk^+^ (**F**) or CD8^+^ T cells (**G**) on week 6. (**H**) Separate experiment wherein serum human IgG^+^ NSG+hIL6 mice were given 4 doses (black arrowheads) of saline (black, *n* = 3, 10 μL/g) or bortezomib (red, *n* = 3, 1 mg/kg i.v.) over 4 weeks. Statistics were calculated with 2-tailed Mann-Whitney *t* tests.

To test the utility of NSG+hIL6 mice for modeling MM therapies, we treated myeloma-engrafted NSG+hIL6 mice with either human BCMA-directed CAR T cells (BCMA-CART cells) (28, 29) or bortezomib. For the BCMA-CART studies, hosts were engrafted with BM cells from a newly diagnosed patient 14 weeks before CAR T cell inoculation, and all hosts possessed human serum IgG within 5 weeks after engraftment. Each host received 3 × 10^5^ cells per dose of untransduced (UTD) or BCMA-CART CD8^+^ T cells from the same normal donor at “week 0,” and serum human Ig titers traced weekly over the subsequent 6 weeks. Whereas serum IgG levels continued to increase in UTD controls, delivery of BCMA-CART cells coincided with an overall decrease in Ig levels to below detection levels in 5 of 6 hosts within 2 weeks of BCMA-CART transfer (Figure 8A), and an overall relative loss in serum Ig levels compared with UTD controls in every host (Figure 8, B and C). Furthermore, BM Igκ^+^ MM cells were also significantly depleted in all BCMA-CART cell–treated mice (Figure 8, D and F), and as anticipated human CD8^+^ T cells were readily detected in all hosts (Figure 8, E and G).

Additionally, 2 separate groups of myeloma-engrafted mice were treated with saline or bortezomib subcutaneously at 1 mg/kg weekly for 4 weeks beginning 30 weeks after transfer of patient BM cells. Here, we employed a dosing schedule and dose roughly equivalent to a standard 1 cycle of therapy used for human MM patients. Upon following serum Ig titers weekly for 6 weeks, we observed that bortezomib significantly decreased titers of human IgG compared with saline controls (Figure 8H). We conclude that NSG+hIL6 mice are a highly suitable model system for study of both cellular therapy and small molecule drug candidates in malignant human plasma cells.

## Discussion

Our results establish that NSG+hIL6 mice with busulfan conditioning are highly suited for the routine and reproducible engraftment, persistence, and progressive growth of patient-derived malignant plasma cells. Supporting this conclusion, NSG+hIL6 mice were readily engrafted with Ig light chain–restricted plasma cells from newly diagnosed and postrelapse myeloma patients as well as donors experiencing MGUS or diagnosed with other plasma cell–driven afflictions, including PCL and AL amyloidosis. Furthermore, with time, mouse recipients of myeloma cells experienced progressive increases in human IgG in serum, and many experienced elements of advanced MM such as anemia, hypercalcinemia, bone lesions, and hind limb paralysis consistent with vertebral involvement and cachexia.

Past work has shown that preestablished myeloma cell lines grow rapidly after transfer into NSG mice, often resulting in rapid dominance of host BM within 4 weeks and death soon thereafter (11). By contrast, in NSG+hIL6 mice, patient-derived myeloma cells often comprised a relatively small fraction of all BM cells and appeared to expand relatively slowly, with a median overall survival of 42 or more weeks. Consistent with this conclusion, only small frequencies of Ki67^+^ cells were observed among implanted myeloma cells. The relatively slow growth rates of engrafted plasma cells and the extended survival times of NSG+hIL6 mice are consistent with human disease (21). Indeed, previous attempts to quantify cell division rates for patient myeloma cells suggest relatively slow doubling times, ranging from weeks to several months (30, 31). Given that unsorted patient BM mononuclear cells were used for engraftment, we speculate these results suggest that supporting cells may be required for PCD growth in human BM and are either slow growing or altogether absent in many engrafted NSG+hIL6 mice. This hypothesis is further supported by the apparent lack of complete BM replacement in NSG+hIL6 hosts. scRNA-seq of the BM confirmed the presence of the original patient myeloma clone as well as the presence of T cells and mast cells. No other human cell types were detectable by transcripts. Given the presence of mast cells almost a year after myeloma cell engraftment, but a lack of other human myelopoiesis (neutrophils, macrophages in particular), there are likely common myeloid progenitors skewed to mast cell differentiation that were not readily able to be distinguished from the whole human mast cell pool. The presence of T cells likely also indicates a potential imperfect depletion by OKT3 rather than repopulation by human CD34^+^ stem-like cells, but either way, their level or function was sufficiently low that GvHD was not observed. Future serial transplantation studies using NSG+hIL6 transgenic hosts may resolve these issues.

Additional facets of the NSG+hIL6 system are also consistent with human MM. In this regard, we note that disparate clinical phenotypes often developed among cohorts of NSG+hIL6 hosts despite receiving identical doses of donor BM cells on the same day from the same myeloma patient. Indeed, some animals took upwards of 6 months before showing detectable antibody in the blood and became moribund soon thereafter, whereas others harbored readily detectable human IgG titers for months before experiencing clinical symptoms. One possible technical reason is varying amounts of IL-6 between different mice. We neither tracked IL-6 levels during experiments nor checked IL-6 levels before transplantation of myeloma cells. There is also the possibility that varying phenotypes are not related to IL-6 levels. Human myeloma phenotypes are similarly variable. In this regard, it remains unknown why certain patients develop certain elements of the disease or why some patients’ disease remains stable for many years before relapsing while others rapidly progress. Ultimately, our model may provide insights into this problem, thereby leading to a better understanding of how myeloma causes complex clinical phenotypes.

With NSG+hIL6 mice, we were able to engraft a diverse set of PCDs in more than 70% of animals (100% of healthy animals) from both fresh and frozen samples at 5–10 weeks after injection, as compared with NSG mice lacking the human *IL6* locus. We used death as a readout, which has seldom been done with past myeloma models, and note that many mice also developed hind limb paralysis at high rates consistent with vertebral involvement and cachexia. Furthermore, longitudinal assay of blood for human antibody titers proved a feasible approach for inferring ongoing treatment response to bortezomib and BCMA-CART cell treatment. Further delineation of what cells are responsible for what clinical effects of these and other drugs could lead to development of supportive therapies that prevent myeloma complications in the future.

In summary, we present a patient-derived xenograft model for PCDs characterized by fidelity to human disease and ease of use. In line with the findings with the MISTRG6 mouse (15), we note dissemination of tumor, circulating disease only with hosts given PCL, and a supportive environment for PCDs in general. The addition of the NSG+hIL6 model and its availability within the research toolbox will aid investigators in the wider PCD research community in the quest for truly durable, curative therapies.

## Methods

### Sex as a biological variant.

Both sexes of mice and patients from which BM samples were procured were used in experiments.

### NSG+hIL6 mice.

NSG+hIL6 transgenic mice (stock 028655) were imported and are available from Jackson Laboratories. A transgenic construct containing the human *IL6* gene from the 161-kb BAC clone RP11-469J8 (BACPAC Resource Center) was microinjected into the pronuclei of fertilized NOD.CB17-*Prkdc^scid^*/J eggs. Founder line 1 was established and mated to to NOD.Cg-*Prkdc^scid^* *Il2rg^tm1Wjl^*/J (stock 005557, Jackson Laboratories) mice, and progeny were interbred until all offspring were homozygous for *scid*, hemizygous/homozygous for the *Il2rg* targeted mutation, and hemizygous for the transgene. All subsequent breeding involved heterozygous males and wild-type females, because female NSG+hIL6 mice have low fertility. All mice were bred and maintained under strict clean conditions to minimize risk of infection per protocols within the Penn Stem Cell and Xenograft Core Facility. PCR genotyping for the hIL6 BAC was performed by Transnetyx using the following oligonucleotides: F-GGGAGAGCCAGAACACAGA and R-TGCAGCTTAGGTCGTCATTG.

### Preparation of primary human cells.

All reagents were dedicated to plasma cell isolation to minimize contamination risk. All parts of this procedure except spinning were done in a tissue culture hood with sufficient laminar air flow. Two to 5 mL of BM aspirate was obtained in green-top heparin tubes (no EDTA). Aspirate was diluted to 16 mL in Dulbecco’s PBS (DPBS) with calcium and magnesium (Thermo Fisher Scientific) in a sterile 50 mL conical tube. Four milliliters of Ficoll Paque plus (Sigma-Aldrich) was carefully added to the bottom of two 15 mL conical tubes, and then diluted aspirate was carefully layered over the Ficoll. After equally distributing 8 mL of diluted aspirate atop each 4-mL Ficoll cushion, tubes were carefully capped and moved to a room temperature swinging bucket centrifuge and spun at 700*g* for 20 minutes without braking. Buffy coats from both tubes were combined into one 50 mL conical tube. Ten milliliters of DPBS with calcium was added and then mixed with inversion before spinning down at 400*g* for 5 minutes with normal braking parameters. Supernatant was removed and 5 mL of ACK lysis buffer (Thermo Fisher Scientific) was added. Samples were pipetted up and down and allowed to lyse at room temperature for 5 minutes. Cells were spun down and supernatant removed. Cells were resuspended in 1 mL of DPBS, mixed with gentle pipetting until single-cell suspensions were obtained, and then counted. Total BM mononuclear cells were used for transplantation. If total cell counts were in the millions, cells were frozen or proceeded directly to transplantation. To freeze cells, BM mononuclear cells were counted, spun, and resuspended in 1 mL cold fetal bovine serum with 10% DMSO in aliquots of 1 × 10^6^ to 5 × 10^6^ cells. Vials were placed in a Corning CoolCell LX container overnight at –80°C, and the next day samples were moved to a liquid N_2_ dewar.

For transplantation, Primocin (Invivogen) was added to the 1 mL cell suspension at 100 μg/mL along with OKT3 antibody at 10 μL/10^6^ cells and incubated at 4°C for 1 hour, as described previously (32). Antibiotics and OKT3 treatment were performed to decrease risk of infection from donor pathogens into immunodeficient animals and to deplete GvHD-causing T cells, respectively. OKT3 does not deplete all T cells, but does prevent GvHD in this model system. A 100 μL aliquot was removed and placed at –20°C for subsequent pathogen testing (IDEXX hIMPACT panel). Remaining cells were spun down and supernatant removed. Cells were diluted to 1 × 10^6^ cells/10 μL/mouse with an extra 10 μL overall to account for loss. In small cohorts of mice, there were no differences noted between transplantation of 5 × 10^5^, 1 × 10^6^, or 2 × 10^6^ mononuclear cells, with a standard dose of 1 × 10^6^ cells. Cells were transplanted within 4 hours of cell preparation completion.

### Xenograft transplantation.

Mice were conditioned with 1 intraperitoneal injection of busulfan (30 mg/kg) 24 hours prior to introduction of prepared patient BM aspirate. Intraosseous injection of aspirate began with anesthetizing mice using isoflurane on anesthesia nose cone. The injection site used was always the left hind limb. The site was shaved just prior to injection and wiped clean 3 times using chlorhexidine wipes. Meloxicam or Meloxicam SR was injected prior to incision. The mouse’s leg was stabilized in a bent position to allow access to the patellar surface of the femur. A hole was punched through the patellar surface into the shaft of the bone using a 25-gauge needle and then a 30-gauge needle was inserted into the femur. An infusion of 10 μL of cells (1 × 10^6^ cells/mouse) was administered using a small-volume syringe. A drop of Vetbond (3M, 14690) was placed at the insertion site when the needle was withdrawn from the femur. Animals were monitored daily for weight loss, malaise, tumors, and limb paralysis. Intravenous injection of patient mononuclear cells was not specifically studied, but preliminary experiments suggest the intraosseous route to be more reliable than intravenous injection.

### Following engraftment markers.

Blood was the easiest and most reproducible way to follow engraftment of malignant plasma cells. The NSG mouse has no antibodies at baseline, mouse or human. By following the increase in human titers of total Ig by ELISA, it was possible to determine which animals had been engrafted and which had not by approximately 5 weeks. In high burden states such as PCL, anticoagulated blood is stainable for malignant cells as well. Blood was collected in Eppendorf tubes and allowed to clot for 30 minutes prior to spinning at 8000*g* for 8 minutes. Serum was then removed to a new tube, leaving red cells behind. Serum was then applied to blood and urine ELISA and SPEP.

### ELISA.

ELISA plates (Thermo Fisher Scientific) were coated using 100 μL/well coating buffer (2.93 g/L NaHCO_3_, 1.59 g/L Na_2_CO_3_; pH 9.6) and 1 μg/mL of unlabeled total anti–human total Ig (Southern Biotech, 2010-01) overnight at 4°C or at 37°C for 1 hour. Wells were then washed with wash buffer 3 times (1× PBS with 0.1% Tween 20). Blocking buffer (0.22 μm–filtered 2% BSA in 1× PBS) was added at 100 μL/well and allowed to block at room temperature for 1 hour. One microliter of serum from each mouse was added to a single well at the top of a column. Samples were then serially diluted 1:10 down the columns 3 times for a total of 4 wells per samples. This allowed for 24 samples to be run on 1 plate. Sera were incubated for 1 hour. Wells were again washed 3 times with wash buffer. Capture buffer (blocking buffer with 1 μg/mL biotin-labeled anti–human total Ig) was added to each well at 100 μL/well. The plate was incubated at room temperature for 1 hour and then washed again 3 times. Streptavidin-HRP (1 μL/10 mL) was added to each well at 100 μL/well and incubated in the dark at room temperature for 1 hour. Wells were again washed 3 times and the plate blotted forcefully against paper towels to remove as much wash buffer as possible. Room temperature TMB substrate (Thermo Fisher Scientific) was prepared and 100 μL added to each well. After wells started turning yellow (1–2 minutes or less), the reaction was quenched with 200 μL of 1 M phosphoric acid. The plate was then assessed for absorbance on a SpectraMax microplate reader (Molecular Devices) at 450 nm, with background subtraction at 570 nm. For quantification, Ig kappa or lambda monoclonal protein (Thermo Fisher Scientific) was run at known concentrations at 10-fold dilutions starting at 1000 ng down an entire column (7 dilutions × 2 columns). Antibody concentrations were determined using 4PL regression in GraphPad Prism 9.

Urine testing for the presence of Ig was also conducted by ELISA with the same method outlined above. Urine was loaded at 10 μL into 100-μL wells before dilution due to lower concentration of Ig. ELISA testing for hIL-6 was carried out with an hIL-6 kit (R&D Systems, DY206).

### Histology/immunohistochemistry.

Tissues were isolated after euthanasia and placed in 10% formalin overnight at 4°C. The next day, fixed tissues were removed to 2 cassettes per mouse, 1 for soft tissues and 1 for bones. These cassettes were then placed in 70% ethanol/30% water and allowed to soak prior to processing. We utilized the histology services of the University of Pennsylvania veterinary school for standard practices in decalcification, paraffin block embedding, tissue slice preparation, and H&E staining. Slices were put on ProbeOn (Thermo Fisher Scientific) slides for immunohistochemistry (IHC).

IHC was performed as per IHC protocol (Abcam). After deparaffinization, slides were submitted to sodium citrate buffer antigen retrieval for 30 minutes prior to overnight incubation of primary antibodies (see Supplemental Table 1; supplemental material available online with this article; https://doi.org/10.1172/jci.insight.177300DS1). The endogenous peroxide background was reduced by incubating the slides in 3% hydrogen peroxide for 10 minutes before incubation of secondary HRP-conjugated antibody and subsequent DAB substrate application for 12 minutes.

### scRNA-seq of myeloma-engrafted BM.

BM cells from an NSG+hIL6 mouse engrafted with human myeloma were fixed and stored at –80°C with the Evercode cell fixation kit v2 from Parse Biosciences. Just prior to processing, cells were thawed and prepared using the Evercode WT mini v2 kit and associated protocol. This is a plate-based barcoding method to perform scRNA-seq. Two sublibraries were generated, one with 5000 cells and the second with 10,000 cells. Sublibraries were submitted to Azenta Life Sciences for sequencing at equimolar ratios on an Illumina NovaSeq 6000 with paired-end 150-bp reads (~350 × 10^6^ reads). Analysis was performed using the Parse Biosciences platform based in R/Python (https://www.parsebiosciences.com).

### Assaying serum ionized calcium level.

Mouse blood was collected in polypropylene 1.2 mL centrifuge tubes without anticoagulants and allowed to clot for 30 minutes prior to spinning at 8000*g* for 8 minutes and transferring the sera to a new tube. Sera were then tested for ionized calcium concentration using the Calcium Assay Kit (Abcam, 102505).

### SPEP.

SPEP was carried out using the QuickGel station from Helena Laboratories and the Split-Beta SPE Kit (3550T) per manufacturer’s instructions.

### Complete blood counts.

At 15 weeks after injection of patient samples from MM1 and MM2, blood was collected in EDTA-coated Vacutainer tubes and sent to IDEXX Analytics for formal complete blood count testing (test code 375).

### MicroCT scanning.

With the help of the Small Animal Imaging Facility Core Resource at the University of Pennsylvania, mice were anesthetized using inductive isoflurane and then maintained through nose cone prior to mounting on the MILabs U-CT ultra-high-resolution (~20 μm) small-to-medium sized animal CT scanner. Four-minute scans were obtained prior to euthanasia. Images were analyzed using ImageJ (NIH).

### Flow cytometry.

Cells were isolated from femurs and spleens on ice, lysed for red blood cells using ACK lysis buffer for 5 minutes at room temperature, and then stained for live cells with Zombie Aqua Live/Dead (Thermo Fisher Scientific) (10 minutes) and fluorescently labeled antibodies of markers of interest (30 minutes) in 0.1% BSA in PBS. Please see Supplemental Table 1 for antibodies used.

### BCMA-CART cells.

BCMA-CART cells were provided by Avery Posey and Michael Milone’s labs at the University of Pennsylvania. The BCMA single chain variable fragment employed for BCMA-CART cells was also used in a clinical trial for relapsed refractory myeloma (29) and consists of Ig heavy and light chain variable regions derived from a BCMA-reactive antibody (clone 10) assembled with an extracellular hinge and transmembrane region derived from CD8 linked to an intracellular signaling cassette derived from CD3ζ and a 4-1BB intracellular domain, as described previously (28). PCR-amplified CAR constructs were subcloned into the pTRPE vector before packaging into lentivirus using a VSVG envelope and HEK293T cells. Patient T cells were stimulated and treated with CAR-containing lentivirus, and then expanded and harvested for injection at 3 × 10^5^ cells/mouse.

### Statistics.

Two-sided ANOVA and appropriate single- or multiple-comparison *t* tests were used and calculated with GraphPad Prism. Specific tests are noted in figure legends. All summary data points are means, and all error bars denote standard deviation. The significance cutoff was an α value of 0.05. A *P* value of less than 0.05 was considered significant. Cohorts MM1 and MM2 were powered at 80% under the assumption that NSG+hIL6 mice would have an engraftment incidence of 80% based on observed engraftment in the MISTRG6 model versus 20% in NSG mice from our prior experience. Treatment with bortezomib could not be powered to the same level given the lack of available myeloma-engrafted mice at the time of experimentation. Unless otherwise stated in figure legends, all displayed experiments were performed once but used only biological replicates (each data point represents a unique mouse).

### Study approval.

All human samples were collected after obtaining informed consent per approved IRB protocol no. 842940 through the PCD group at the Hospital of the University of Pennsylvania. All mouse experiments were performed under the stem cell and xenograft core IACUC protocol for animal model development. Humane endpoints were used to determine when mice were euthanized. These included weight loss greater than 20%, hind limb paralysis, extreme lethargy, and respiratory distress.

### Data availability.

scRNA-seq data have been uploaded to the NCBI Gene Expression Omnibus database (GEO GSE246140, GSM7857100, and GSM785710). Both raw data files and normalized files from which the analyses within this manuscript were derived are available for download. Code for scRNA-seq analysis is available from Parse Biosciences. A Supporting Data Values file for all figures is available in the supplemental material.

## Author contributions

ZSH designed and performed experiments, analyzed data, and wrote the manuscript. ALG provided patient samples and wrote the manuscript. LB, LDS, YT, SK, WL, and NCS designed and performed experiments. DD performed experiments. DTV, ADC, AJW, and SPS provided patient samples and reviewed the manuscript. MC and EAS designed experiments and wrote the manuscript. DA designed experiments, analyzed data, and wrote the manuscript.

## Supplementary Material

Supplemental data

Supporting data values

## Figures and Tables

**Table 1:**
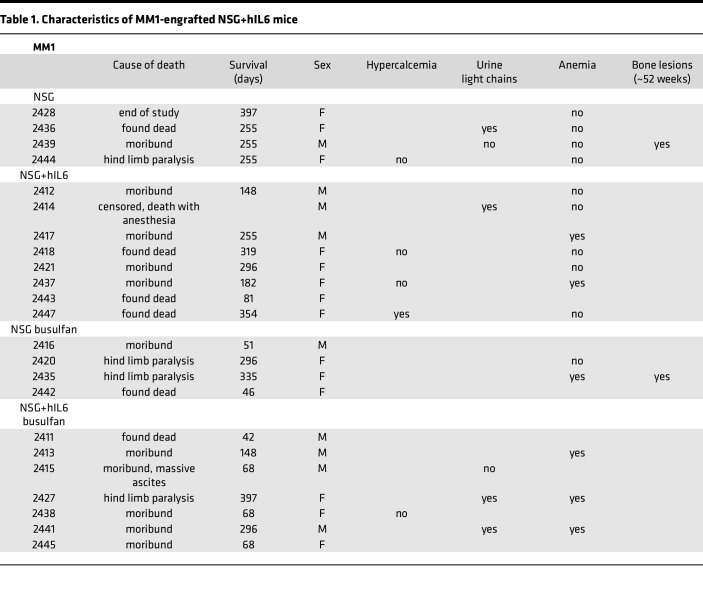
Characteristics of MM1-engrafted NSG+hIL6 mice

**Table 2:**
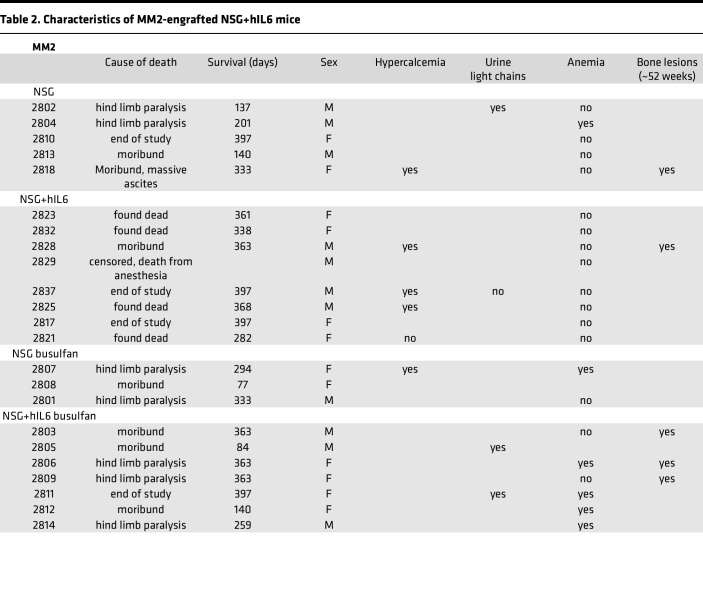
Characteristics of MM2-engrafted NSG+hIL6 mice

## Version 1 05/07/2024

In-Press Preview

## Version 2 05/22/2024

Electronic publication
