# Supplementary material for: Human IL-6 fosters long-term engraftment of patient-derived disease-driving myeloma cells in immunodeficient mice
Source: JCI Insight. 2024 May 7;9(10):e177300. doi: 10.1172/jci.insight.177300 (PMC11141932; doi:10.1172/jci.insight.177300)
Supplement: Supplemental data [file jciinsight-9-177300-s164.pdf]

Supplemental Information:

Supplemental Table1: Reagent Specifics

	Vendor	Product ID	Assay
Primers			
F-GGGAGAGCCAGAACACAGA	transnetyx	hIL-6	genotyping
R-TGCAGCTTAGGTCGTCATTG	transnetyx	hIL-6	genotyping
Antibodies			
OKT3	Thermo	16-0037-81	T-cell depletion
anti human total Ig - unlabelebd	Southern Biotech	2010-01	ELISA
anti human total Ig-Biotin	Southern Biotech	2010-08	ELISA
anti human IgG - Biotin	Southern Biotech	2040-08	ELISA
anti human IgM - Biotin	Southern Biotech	9020-08	ELISA
anti human IgA - Biotin	Southern Biotech	2050-08	ELISA
sterptavidin-HRP	Biolegend	405210	ELISA
mouse anti human CD138	BioRad	MCA2459T	IHC
mouse anti human kappa	BioRad	5268-6010	IHC
anti mouse Ig HRP	Biolegend	405306	IHC
anti mouse CD45.1	Biolegend	110703	Flow cytometry
Streptavidin BUV 661	BD biosciences	612979	Flow cytometry
anti human CD138 BUV737	BD biosciences	612834	Flow cytometry
anti human CD19 BUV805	BD biosciences	742007	Flow cytometry
anti human CD38 BV421	Biolegend	356617	Flow cytometry
LiveDead Aqua	Thermo	L34957	Flow cytometry
anti human CD47 BV605	Biolegend	323119	Flow cytometry
anti human CD20 BV650	Biolegend	302335	Flow cytometry
anti human Ki67 BV711	Biolegend	350515	Flow cytometry
anti human CD27 BV785	Biolegend	302831	Flow cytometry
anti human IgL AF488	Southern Biotech	9180-30	Flow cytometry
anti human CD45 PerCP-Cy5.5	Biolegend	368503	Flow cytometry
anti human CD269 (BCMA) PE	Biolegend	357503	Flow cytometry
anti human CD24 PE-CF594	BD biosciences	562405	Flow cytometry
anti human CD200 PE-Cy7	Biolegend	329211	Flow cytometry
anti human IgK APC	Southern Biotech	9230-11	Flow cytometry
anti human CD56 AF700	Biolegend	362521	Flow cytometry
anti human CD14 APC-Cy7	Biolegend	367107	Flow cytometry
